# Efficacy of nano‐particulated, water‐soluble erlotinib against intracranial metastases of EGFR‐mutant lung cancer

**DOI:** 10.1002/1878-0261.12394

**Published:** 2018-11-02

**Authors:** Dong Ha Kim, Yun Jung Choi, Ki Jung Sung, Seon‐A Yoo, Young Hoon Sung, Jeong Kon Kim, Chang‐Min Choi, Miyong Yun, Eun Yong Lee, Yong Suk Jin, Seungho Cook, Jin Kyung Rho, Jae Cheol Lee

**Affiliations:** ^1^ Department of Pulmonology and Critical Care Medicine Asan Medical Center College of Medicine University of Ulsan Seoul South Korea; ^2^ Asan Institute for Life Sciences Asan Medical Center College of Medicine University of Ulsan Seoul South Korea; ^3^ Department of Convergence Medicine Asan Medical Center College of Medicine University of Ulsan Seoul South Korea; ^4^ Department of Radiology Research Institute of Radiology Asan Medical Center College of Medicine University of Ulsan Seoul South Korea; ^5^ Department of Oncology Asan Medical Center College of Medicine University of Ulsan Seoul South Korea; ^6^ Department of Bioindustry and Bioresource Engineering College of Life Sciences Sejong University Seoul South Korea; ^7^ Bio‐Synectics, Inc. Seoul South Korea; ^8^ Biomedical Sciences College of Medicine University of Ulsan Seoul South Korea

**Keywords:** brain metastasis, EGFR‐TKI, lung cancer, nano‐particulation, NUFS‐sErt

## Abstract

Central nervous system (CNS) metastasis is one of the serious complications of epidermal growth factor receptor (EGFR)‐mutant lung cancer, which arises due to poor penetration of the brain–blood barrier by EGFR‐tyrosine kinase inhibitors (EGFR‐TKIs). Although osimertinib, a third‐generation EGFR‐TKI, has efficacy against CNS metastases, further treatment modalities are still needed as some of these lesions do not respond to osimertinib, or undergo progression after an initial response to this drug if radiotherapy has already been conducted. Here, we investigated the efficacy of water‐soluble erlotinib (NUFS‐sErt) against these metastases. This agent was synthesized using a nano‐particulation platform technology utilizing fat and supercritical fluid (NUFS™) to resolve the low solubility problem that typically prevents the creation of injectable forms of EGFR‐TKIs. The average NUFS‐sErt particle size was 236.4 nm, and it showed time‐dependent dissolution in culture media. The effects of NUFS‐sErt were similar to those of conventional erlotinib in terms of inhibiting the proliferation of EGFR‐mutant lung cancer cells and suppressing EGFR signaling. In an intraperitoneal xenograft model of HCC827 cells, intraperitoneal administration of NUFS‐sErt produced a dose‐dependent inhibition of tumor growth and enhanced survival rate. Notably, the injection of NUFS‐sErt into the brain ventricle caused significant tumor growth inhibition in an intracranial xenograft model. Hence, our current findings indicate that NUFS‐sErt is a novel, water‐soluble form of erlotinib that can be administered using intraventricular or intrathecal injections. The target cases would be patients with a progressive CNS metastasis and no other therapeutic options. This drug could also be given intravenously to patients with swallowing difficulties or an inability to ingest due to a medical condition.

**Abbreviations**
BBB: brain–blood barrier
CNS: central nervous system
CSF: cerebrospinal fluid
EGFR‐TKI: epidermal growth factor receptor‐tyrosine kinase inhibitor
NSCLC: non‐small‐cell lung cancer
NUFS: Nano‐particulation using fat and supercritical fluid
NUFS‐sErt: NUFS‐soluble erlotinib
PFS: progression‐free survival
RR: response rate

## 1 Introduction

The central nervous system (CNS), including the brain and leptomeninges, is a common site of metastasis from non‐small‐cell lung cancer (NSCLC). CNS metastases cause a rapid deterioration in the patient's health and a poor survival outcome (Ceresoli *et al*., 2002). Epidermal growth factor receptor‐tyrosine kinase inhibitors (EGFR‐TKIs) have shown some efficacy in patients with CNS metastases from an EGFR‐mutant lung cancer (Economopoulou and Mountzios, 2016; Park *et al*., 2012; Wu *et al*., 2013). However, most of these responsive patients eventually experience CNS failure within a certain period and this is more prevalent in patients who show a clinical benefit from EGFR‐TKI therapy (Lee *et al*., 2010; Omuro *et al*., 2005). The improvement in survival caused by EGFR‐TKIs in patients with an EGFR‐mutant lung cancer, and the propensity of these cancers to lead to a metastatic brain tumor, may contribute to this phenomenon (Matsumoto *et al*., 2006). However, persistent control of extra‐cranial site metastases has been described in many patients indicating that incomplete penetration of the brain–blood barrier by EGFR‐TKIs may be the main cause of CNS failure in lung cancer cases (Lee *et al*., 2010; Park *et al*., 2012).

At present, a third‐generation, mutant‐selective EGFR‐TKI, osimertinib, is clinically available to treat T790M‐mediated resistance. It also has a higher brain penetration rate which results in good efficacy in the CNS. In a preclinical study in the mouse brain, the brain/plasma C_max_ ratio of this agent was measured at 3.41, which was much higher than that of gefitinib, afatinib, and rociletinib (0.21, <0.36, and <0.08, respectively) (Ballard *et al*., 2016). In previous subgroup analysis from a phase III AURA3 trial, the CNS response rate (RR) and progression‐free survival (PFS) outcomes in patients with measurable CNS lesions were 70% and 11.7 months with osimertinib, compared with 31% and 5.6 months with chemotherapy, respectively (Mok *et al*., 2017). The clinical outcomes of patients with a CNS metastasis from an EGFR‐mutant lung cancer have thus improved with the emergence of osimertinib. However, issues still exist with treatments for these tumors as some patients do not respond to osimertinib and some experience subsequent progression after an initial response to this drug if radiotherapy has already been conducted for the brain. Because osimertinib is a substrate of efflux transporters such as p‐glycoprotein and breast cancer‐resistant protein (Ballard *et al*., 2016), altered expression of these proteins may contribute to later progression although the exact mechanism needs to be explored.

In our present study, we suggest a different treatment approach for CNS metastases that involve a nano‐particulated, water‐soluble EGFR‐TKI which can be delivered via intraventricular or intrathecal routes. Current EGFR‐TKIs have a very low solubility at a neutral pH so that injectable forms of these agents are not feasible. In contrast, NUFS‐sErt was generated using a nano‐particulation platform utilizing fat and supercritical fluid (NUFS™). We believe that this form of EGFR‐TKI could be a viable alternative to oral EGFR‐TKIs, including osimertinib, if they fail to control CNS metastasis.

## 2 Materials and methods

### 2.1 Cell culture and reagents

Human NSCLC cell lines (HCC827 and H1975) were purchased from the American Type Culture Collection (Rockville, MD, USA). The PC‐9 cell line was a kind gift from Kazuto Nishio (National Cancer Center Hospital, Tokyo, Japan). Cells were cultured in 10% fetal bovine serum (FBS), 100 U·mL^−1^ penicillin, and 100 μg·mL^−1^ streptomycin (Invitrogen, Carlsbad, CA, USA) at 37 °C in an atmosphere of 5% CO_2_. All cells were confirmed to be mycoplasma‐free using a MycoProbe Mycoplasma detection kit (R&D Systems, Minneapolis, MN, USA). Erlotinib free base was purchased from Jinan Rouse Industry Co., Ltd. (Jinan, China). Poloxamer 188 and myristyl alcohol (Kolliwax VR, Foster, CA, USA) were purchased from BASF (Florham Park, Morris, NJ, USA). Polyoxyethylene 40 stearate and lecithin (>99%) were purchased from Sigma‐Aldrich, (St. Louis, MO, USA).

### 2.2 Preparation of NUFS‐sErt powder

Nano‐particulation using fat and supercritical fluid‐sErt had been prepared using NUFS™ technology (nanoparticulation using fat and supercritical fluid) from Bio‐Synectics as part of a previous study (Yang *et al*., 2017). One gram of erlotinib free base, 0.2 g of poloxamer 188, and 10 g of myristyl alcohol were mixed and clearly melted at 130 °C. This melted mixture was then rapidly solidified at −4 °C and sieved with 2‐ to 3‐mm mesh to generate a powder. The powder was placed in a pressure‐resistant reactor (BS‐SF‐1; Bio‐Synectics, Inc., Seoul, Korea) and a continuous flow of supercritical fluid (80–90 mL·min^−1^, 17–25 °C, 80–95 atm) removed only myristyl alcohol from the solidified mixture. Ultimately, NUFS‐sErt, which is composed only of erlotinib and the excipients, was prepared.

### 2.3 Preparation of Nano‐sErt (NUFS‐sErt dispersion)

Twenty‐four milligrams of NUFS‐sErt powder (equivalent to 20 mg of erlotinib free base) and 3.2 milliliters of distilled water were mixed in a 10‐mL vial by mechanical shaking for 1 min. Aliquots of 400 μL polyoxyethylene 40 stearate and lecithin solution (each at a 10 mg·mL^−1^ concentration) were then added to the suspension. This mixture was sonicated at 20 °C or below for 15–20 min using a water bath‐type sonicator (KS‐100; Kum Sung Ultrasonic, Seoul, Korea), and ultimately, Nano‐sErt was prepared.

### 2.4 DLS measurements

For particle size analysis, 20 μL of Nano‐sErt was combined with 980 μL of distilled water. The particle size and distribution of this sample were then determined by dynamic light scattering (DLS) using an electrophoretic light scattering spectrophotometer (ELSZ‐1000; Otsuka Electronics Co., Ltd, Osaka, Japan). The solvent used for the particle size analysis was water. The refractory index was 1.3328; the viscosity was 0.8898 cP, and duplicate measurements were taken at 20 °C.

### 2.5 MTT assay

Cells (5 x 10^3^) were seeded into 96‐well plates overnight and then treated with the relevant agents. After 72 h, 15 μL of MTT solution (5 mg·mL^−1^) was added to each well, and the plates were further incubated for 4 h. Crystalline formazan was solubilized with a 100 μL aliquot of 10% (w/v) SDS solution for 24 h. The absorbances at 595 nm were read spectrophotometrically using a microplate reader. Each assay consisted of 8 replicate wells and was repeated at least three times. Data were expressed as the percentage survival compared with the control which was calculated from the absorbance and was background corrected.

### 2.6 Immunoblotting

Cells were lysed in buffer containing 137 mmol·L^−1^ NaCl, 15 mmol·L^−1^ EGTA, 0.1 mmol·L^−1^ sodium orthovanadate, 15 mmol·L^−1^ MgCl2, 0.1% Triton X‐100, 25 mmol·L^−1^ MOPS, 100 mmol·L^−1^ phenylmethylsulfonyl fluoride, and 20 mmol·L^−1^ leupeptin, adjusted to pH 7.2. Lysis of tumor specimens was performed using the Omni Tissue Homogenizer (TH; Omni International, Kennesaw, GA, USA). Antibodies specific for p‐EGFR (Tyr1173), EGFR, Akt, ERK, and actin were obtained from Santa Cruz Biotechnology (Santa Cruz, CA, USA), and antibodies against p‐Akt (Ser473), and p‐ERK (Thr202/Tyr204) were purchased from Cell Signaling Technology (Beverly, MA, USA). Proteins were detected with an enhanced chemiluminescence Western Blotting Kit (Amersham Biosciences, Piscataway, NJ, USA), in accordance with the manufacturer's instructions.

### 2.7 Animal models

The luciferase reporter gene, RedFect lentiviral particle (PerkinElmer, Waltham, MA, USA), was stably integrated into the human NSCLC cell line, HCC827, to generate HCC827‐luc cells for implantation. To establish an intraperitoneal and intracranial xenograft mouse model, female severe combined immunodeficiency (SCID) mice (18–20 g, 6 weeks of age) were purchased from Charles River Laboratories. All experimental procedures were conducted in accordance with a protocol approved by the Institutional Animal Care and Use Committee of Asan Institute for Life Sciences (2017‐14‐271). Mice were each intraperitoneally injected with 10^7^ HCC827‐luc cells to generate the intraperitoneal xenograft model and with 10^5^ of these cells through an intracerebral injection in the case of the intracranial xenograft model as described in a previous study (Rho *et al*., 2017), with minor modifications.

Briefly, mice were anesthetized with 5% isoflurane with oxygen as a carrier gas which was maintained at 2–3% during surgery. The head of the mouse was stabilized using a Harvard Apparatus stereotaxic head frame. After disinfection of the skin, a 1‐cm midline scalp incision was made, and two burr holes (coordinates of tumor implantation, 2.5 nm lateral, 0.5 nm posterior to the bregma, and 3.5 depth; coordinates of intrathecal injection, 1.1 nm lateral, 0.5 nm posterior to the bregma, and 2.5 mm depth) were made in the skull using a high‐speed microdrill. Tumor cells were injected into the left striatum using a 10‐μL Hamilton syringe. A screw device was then fixed into the right ventricle of the brain. The Burr holes in the skull were sealed with bone wax, and the incision was closed using Dermabond. To confirm that the stereotaxic coordinates used throughout the study were suitable for NUFS‐erlotinib injection into lateral ventricles, Evans blue was injected into these ventricles via an intrathecal catheter and the presence of blue dye in the surrounding brain tissue was examined 10 min after injection. Tumor growth *in vivo* was monitored and measured via bioluminescence imaging (BLI).

### 2.8 Bioluminescence monitoring

Peritoneal and intracranial tumor growth quantified by BLI was performed using an IVIS spectrum system (Caliper; PerkinElmer Company, Seoul, Korea). Mice were administered an intraperitoneal injection of D‐luciferin (Caliper Life Sciences, Hopkinton, MA, USA) dissolved in DPBS (Invitrogen) at a dose of 150 mg·kg^−1^ body weight. Before and during imaging, mice were anesthetized using 1% isoflurane inhalation (Forane; Arkema, Seoul, Korea). Bioluminescent signals were acquired with an open filter or emission at 620 nm using autoacquisition and a field of view of 13.4 cm. Bioluminescent signals were quantified as the radiance (photon/sec/cm^2^/sr) within a circular region of interest (ROI) using living image 4.4 software (PerkinElmer Company).

### 2.9 Statistics

Data are presented as the mean ± standard deviation. *P* values were determined using unpaired t‐tests between groups using graphpad prism software (GraphPad Software Inc., San Diego, CA, USA).

## 3 Results

### 3.1 Generation of NUFS‐sErt

**Figure 1:**
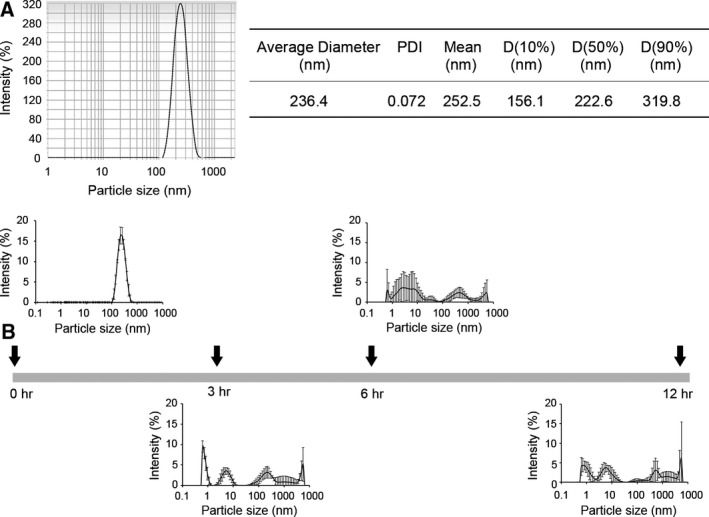
Characterization of NUFS‐sErt. (A) Particle size and distribution of NUFS‐sErt determined by dynamic light scattering (DLS) using an electrophoretic light scattering spectrophotometer. (B) Measured dispersion of NUFS‐sErt in culture media at the indicated times. Error bars are represented as mean ± SD (*n *=* *5).

To improve the solubility of erlotinib, we employed NUFS™technology that was developed to enhance the solubilization of poorly water‐soluble drugs and the bioavailability of these agents through the method of ‘nanoparticulation using fat and a supercritical fluid’ (NUFS) (Park *et al*., 2013). Water‐soluble erlotinib (NUFS‐sErt) was thereby produced, and we confirmed that its average particle size was 236.4 nm. The polydispersity index (PDI) value for NUFS‐sErt was below 0.2, indicating a uniform particle size distribution during water dispersion (Fig. 1A). However, when NUFS‐sErt was added to culture media at 37 °C, a time‐dependent dissolution was evident (Fig. 1B). In addition, NUFS‐sErt has shown an improved solubility and an increased dissolution rate compared with erlotinib in a previous pharmacokinetic (PK) study in dogs, although the formulation of the drug was different in that report due to different administration route requirements (Yang *et al*., 2017).

### 3.2 Efficacy of NUFS‐sErt in EGFR‐mutant NSCLC cells

**Figure 2:**
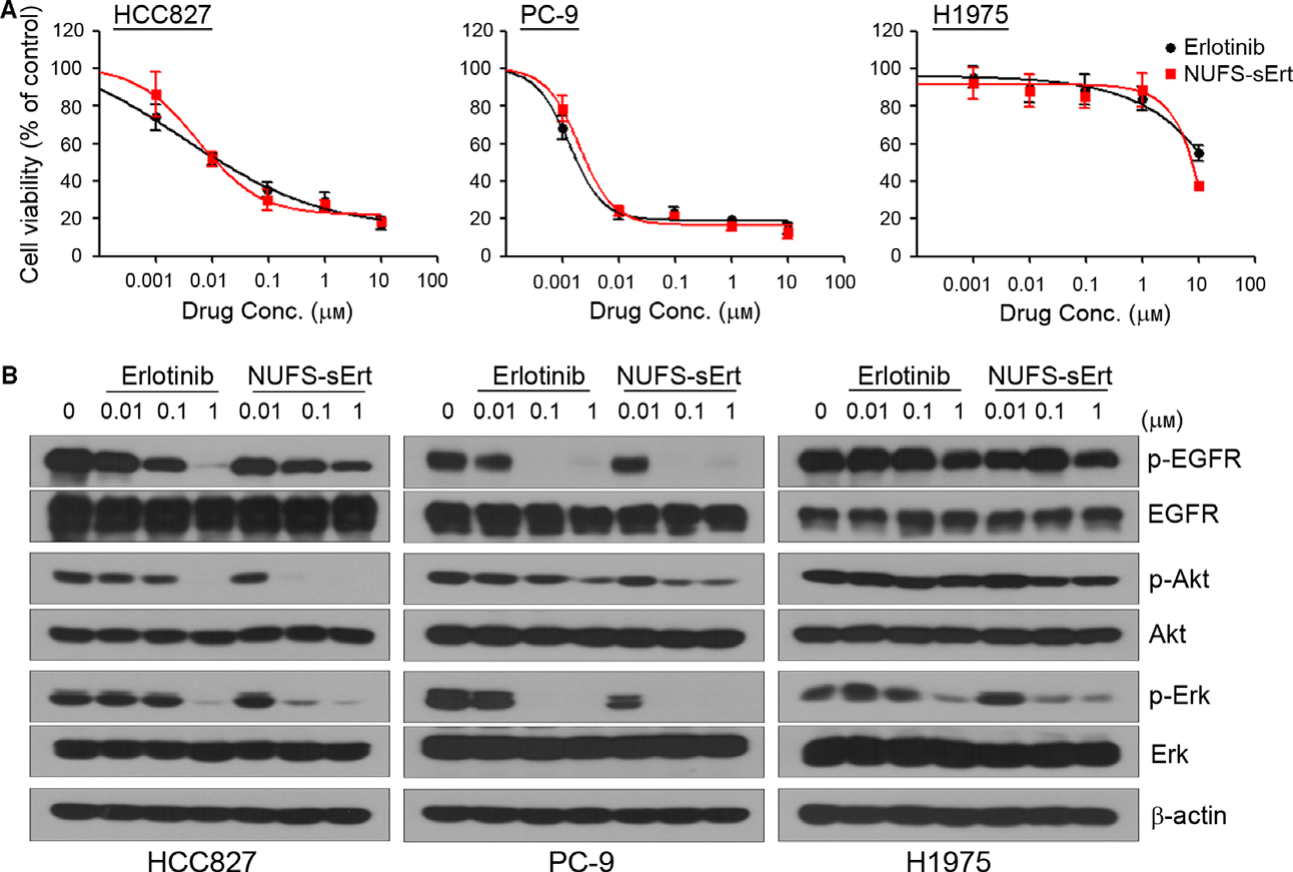
Effects of NUFS‐sErt in mutant‐EGFR NSCLC cells. (A) Cells were treated with the indicated doses of NUFS‐sErt or erlotinib for 72 h, and cell viability was determined using the MTT assay. Error bars are represented as mean ± SD (*n *=* *3). (B) Cells were treated with the indicated doses of NUFS‐sErt or erlotinib for 6 h. Molecules associated with EGFR signaling activity were detected using immunoblotting.

To examine the anticancer activity of NUFS‐sErt and its effects on EGFR‐related signaling in mutant NSCLC cells compared with erlotinib, we performed MTT assays and immunoblotting. As shown in Fig. 2A, NUFS‐sErt treatment was effective against cells with an activating EGFR mutation (HCC827 and PC‐9, exon 19 deletion) but not H1975 cells with a T790M mutation which were resistant to this agent. Consistent with its anticancer properties, NUFS‐sErt also substantially inhibited EGFR activity and its downstream signaling molecules such as Akt and Erk in both HCC827 and PC‐9 cells (Fig. 2B). NUFS‐sErt thus showed similar functional properties to erlotinib. To further validate these findings, we generated another NUFS‐EGFR‐TKI using gefitinib. The resulting water‐soluble gefitinib compound (NUFS‐sGef) also substantially inhibited cell growth without cytotoxic side effects from excipients including polyoxyethylene 40 stearate and lecithin (Fig. S1). Taken together, these data suggest that NUFS‐sErt and other NUFS‐EGFR‐TKI molecules will have the same potency as their conventional drug counterparts.

### 3.3 Efficacy of NUFS‐sErt *in vivo*

**Figure 3:**
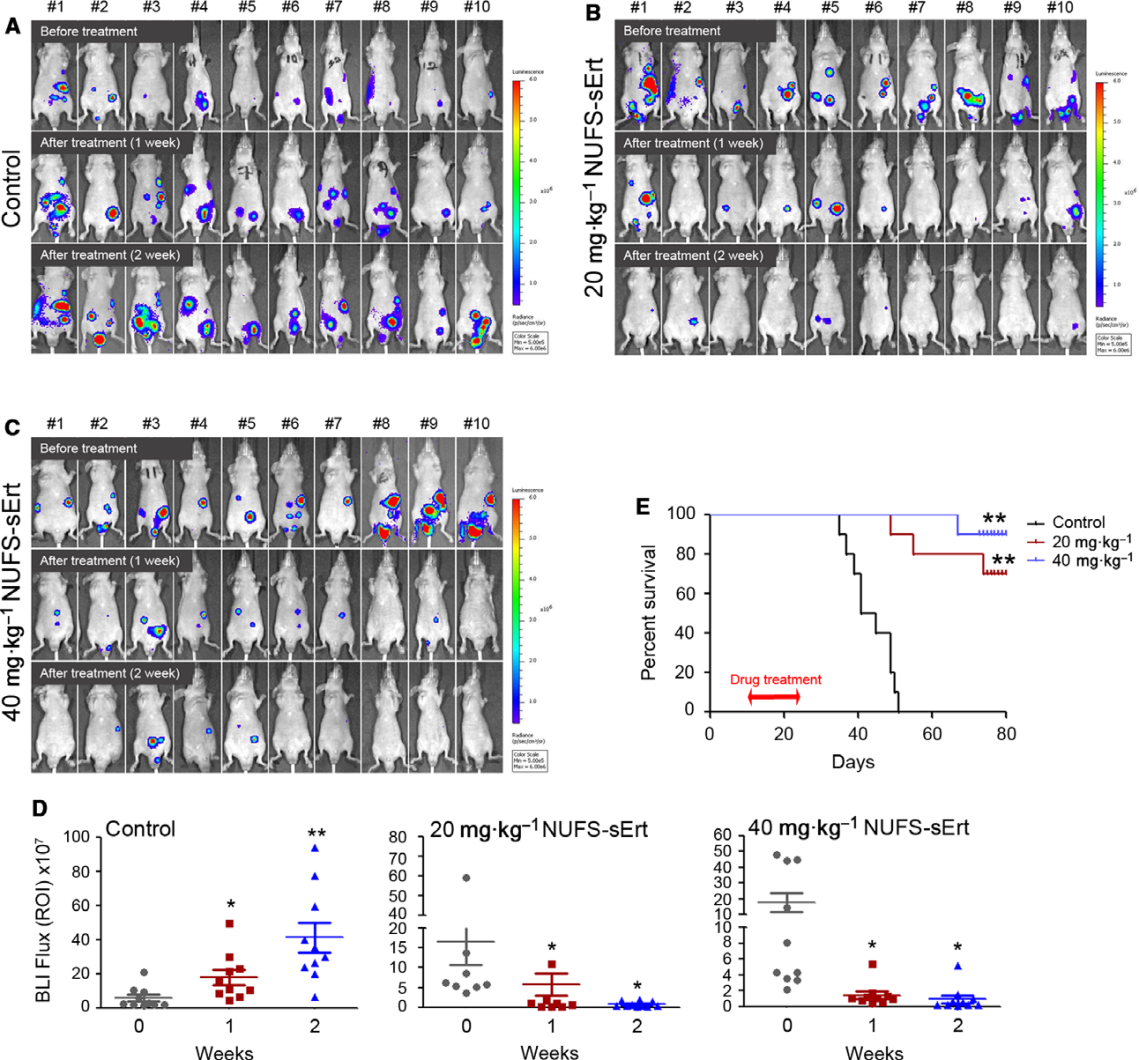
Antitumor activity of NUFS‐sErt in an intraperitoneal xenograft model. Establishment of the EGFR‐mutant lung adenocarcinoma intraperitoneal model using HCC27 cells stably expressing the luciferase reporter (HCC827‐Luc). Bioluminescent imaging (BLI) was used to detect and monitor intraperitoneal tumor growth *in vivo*. (A‐D) BLI images and quantification analysis of intraperitoneal HCC827‐Luc tumor growth before and during treatment with NUFS‐sErt (5 consecutive days/week, *n *=* *10 animals) at the indicated time points and drug doses. Red pseudo‐coloring indicates increased tumor growth, and green‐blue pseudo‐coloring indicates decreased tumor growth by bioluminescence quantification in A–C. (D) Quantification of the bioluminescence photon flux in the mice with intraperitoneal HCC827‐Luc tumors treated over the indicated time points. Error bars are represented as mean ± SD. (E) Kaplan–Meier survival curves of the HCC827‐Luc cell line in mice treated with the indicated doses of NUFS‐sErt for 2 weeks. *P <* *0.01 and **P <* *0.001 for drug versus control (vehicle treated) tumors by Student's *t*‐test. For all treatment studies, baseline imaging and subsequent therapy was initiated 10 days following intraperitoneal tumor cell implantation.

To next examine the effects of NUFS‐sErt *in vivo*, we established an intraperitoneal HCC827‐Luc xenograft model in which we assessed the response to this drug by bioluminescence imaging. The signal intensity in the NUFS‐sErt treatment group was significantly lower than that in the vehicle treatment group (Fig. 3A–D). This tumor growth inhibition was also dose‐dependent, and the tumor signals of some mice had disappeared after 2 weeks of treatment. Consistent with these results, we found also that NUFS‐sErt treatment substantially improved the survival of mice with intraperitoneal EGFR‐mutant tumors (Fig. 3E).

**Figure 4:**
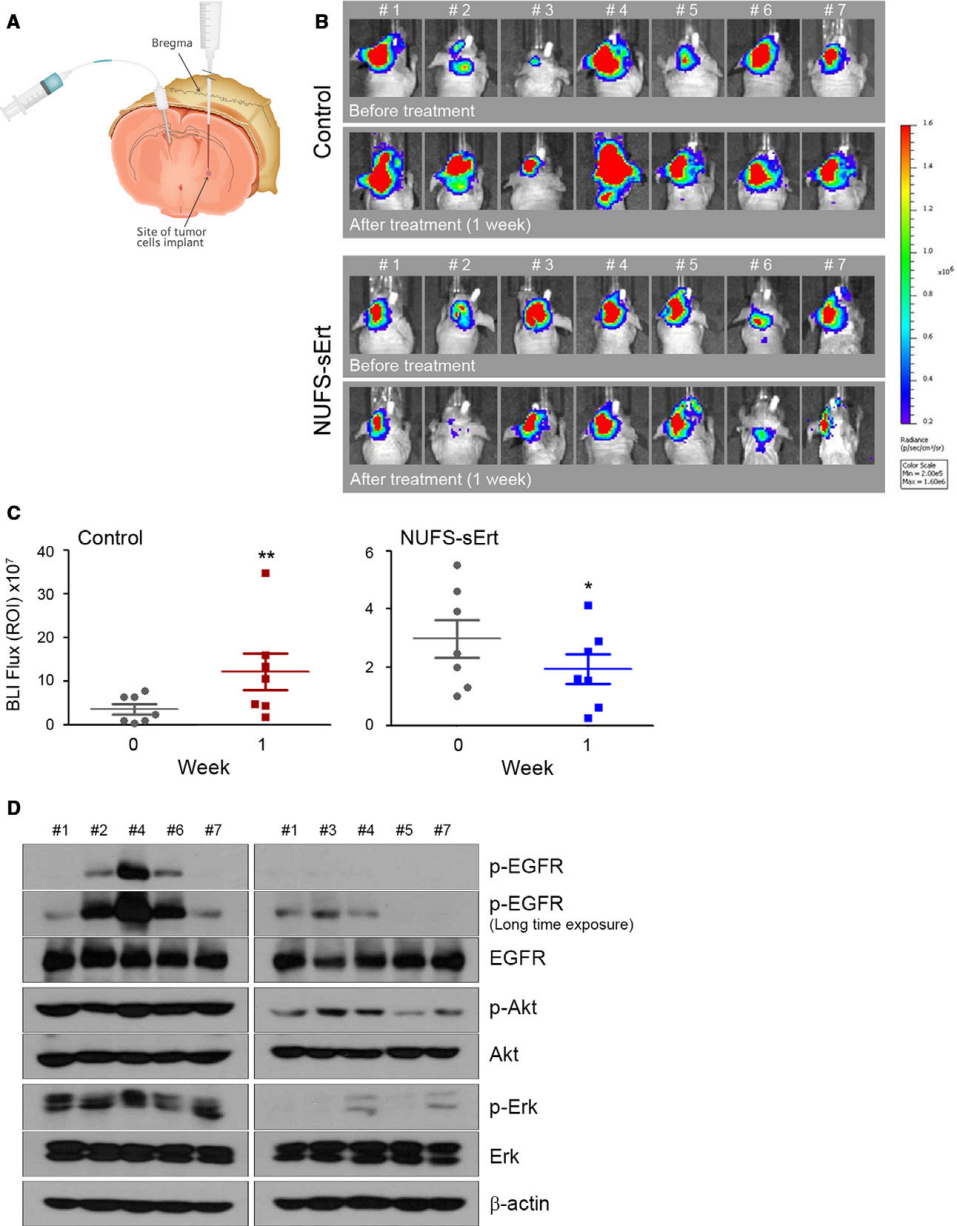
Antitumor activity of NUFS‐sErt in an intracranial xenograft model. (A) Generation of the EGFR‐mutant lung adenocarcinoma intracranial model via intrathecal injections with HCC827‐Luc cells. Cells were injected into the left striatum, and the route of intrathecal injection was fixed into the right ventricle of the brain as described in the Materials and Methods section. (B, C) BLI images and quantification analysis of intracranial HCC827‐Luc tumor growth before and during treatment with NUFS‐sErt (20 μg/4 μL, 2 times/week, *n *=* *7 animals) for 1 week. Red pseudo‐coloring indicates increased tumor growth, and green‐blue pseudo‐coloring indicates decreased tumor growth by bioluminescence quantification in B. (C) Quantification of the bioluminescence photon flux in the mice with intracranial HCC827‐Luc tumors treated over the indicated time points. Error bars are represented as mean ± SD. *P <* *0.01 and **P <* *0.001 by Student's *t*‐test. For all treatment studies, baseline imaging and subsequent therapy was initiated 14 days after intracranial tumor cell implantation. (D) Immunoblot analysis measuring each indicated pharmacodynamics biomarker in representative control‐treated or NUFS‐sErt‐treated tumors harvested from tumor‐bearing mice at 1 week following the initiation of therapy.

We next tested whether the intrathecal injection of NUFS‐sErt could efficiently suppress the growth of intracranial HCC827‐Luc tumors. We established a mouse model with a HCC827‐Luc intracranial xenograft in the left brain, as described previously (Rho *et al*., 2017). We fixed a screwing device into the right brain ventricles of these animals to enable intrathecal drug injection (Fig. 4A). NUFS‐sErt treatment resulted in a marked reduction in tumor proliferation, whereas the vehicle‐treated animals showed enhanced tumor proliferation (Fig. 4B,C; 455 ± 289% with vehicle versus 61 ± 19% with NUFS‐sErt; *P *<* *0.01). All mice of NUFS‐sErt treatment also showed the inhibition of tumor proliferation although the rate of growth inhibition was different. In addition, the significant inhibition of EGFR‐related signaling was observed in mice of NUFS‐sErt treatment (Fig. 4D). Our findings thus collectively suggest the therapeutic potential of NUFS‐sErt to manage the progression of a brain metastasis from an EGFR‐mutant lung cancer.

## 4 Discussion

After proven effects of osimertinib on CNS metastasis, patients with EGFR‐mutant lung cancer acquire another powerful tool to manage this dismal complication. First‐ or second‐generation EGFR‐TKIs such as gefitinib, erlotinib and afatinib have a poor brain penetration rate of only around 1%, leading to a high failure rate of these drugs with CNS lesions (Togashi *et al*., 2012). This problem has also been well documented by Jackman *et al*. (2006). A dose of 500 mg·day^−1^ gefitinib (double the routine dose) reached a level of only 6.2 nm in the cerebrospinal fluid (CSF) of a patient, which is below the effective level required to control tumor cells. Although the effective CSF concentration to achieve a clinical response was achieved with a 1000 mg·day^−1^ dosage in that case, this drug concentration was not tolerable due to side effects such as somnolence and increased hepatic transaminase activity. Hence, the efficacy of osimertinib can be explained by its higher brain penetration rate.

We previously described a third‐generation EGFR‐TKI (GNS compound) which is similar to osimertinib in terms of its effects on EGFR‐mutant cell lines, xenograft models and selectivity of kinases, but showed a superior intracranial antitumor efficacy (Rho *et al*., 2017). In our analyses in that study, osimertinib as well as GNS compound at 10 mg·kg^−1^ showed a good response initially, but this was not sustained over time in most of the mice. We and others have also observed a tumor re‐growth tendency after 50 days following an initial regression, even with a 25 mg·kg^−1^ daily dosage of osimertinib, the exact cause of which requires further investigation (Ballard *et al*., 2016). Hence, although a considerable intracranial response duration can be achieved with third‐generation EGFR‐TKIs, more therapeutic options are going to be eventually needed in most patients.

Recent advances in nanotechnology have enhanced the efficiency of drug delivery by improving the solubility, stability, and biocompatibility of therapeutic compounds. Our nanoparticulation method using fat and supercritical fluid (NUFS™) utilizes supercritical CO_2_ as the extracting solvent (Choi *et al*., 2015). This approach prevents the undesirable side effects of other commonly used excipients and can produce nano‐sized particles in large quantities. Erlotinib has a very low solubility at neutral pH, largely preventing its delivery by injection (Yang *et al*., 2017). We have however applied our NUFS™technology to produce a water‐soluble, nano‐particulated erlotinib, NUFS‐sErt, which showed a comparable efficacy to its parent compound against EGFR‐mutant lung cancer cell lines. Accordingly, the intraperitoneal injection of NUFS‐sErt produced a dose‐dependent inhibition of tumor growth and enhanced survival rate in the mouse. Hence, NUFS‐sErt is an efficacious and safe form of erlotinib that has the distinct advantage of being injectable. This property will be useful for patients with swallowing difficulties or an inability to ingest due to a medical condition. We administrated NUFS‐sErt into the brain ventricle in an intracranial xenograft mouse model and observed significant inhibition of tumor growth *in vivo*. Our current results thus suggest the possible future use of NUFS‐sErt to manage CNS metastases in EGFR‐mutant lung cancer patients, particularly in cases that are not responsive to osimertinib or experience progression after an initial response to radiotherapy.

## 5 Conclusions

Nano‐particulation using fat and supercritical fluid‐sErt is a water‐soluble version of erlotinib generated using a nano‐particulation platform utilizing fat and supercritical fluid to resolve the low solubility problems with the native compound. NUFS‐sErt shows a comparable anticancer potency to erlotinib in mutant‐EGFR NSCLC cells. NUFS‐sErt and NUFS technology may thus serve as new treatment strategies in EGFR‐mutant lung cancer patients with CNS failure and help to resolve some of the drug delivery issues with conventional EGFR‐TKIs.

## 7 Author contributions

JKR, DHK, and JCL conceived and designed the study. YHS, JKK EYL, and YSJ contributed to development of methodology. DHK, YJC, KJS, S‐AY, YHS, EYL, YSJ, C‐MC, SC, and JKR contributed to acquisition of data. DHK, MY, JCL, and JKR analyzed and interpreted data. DHK, JCL, SC, and JKR wrote, reviewed, and revised the manuscript. JCL and JKR supervised the study.

## Supporting information


**Fig. S1.** Effects of NUFS‐sGef in mutant‐EGFR NSCLC cells.

## References

[mol212394-bib-0001] Ballard P , Yates JW , Yang Z , Kim DW , Yang JC , Cantarini M , Pickup K , Jordan A , Hickey M , Grist M *et al* (2016) Preclinical comparison of Osimertinib with other EGFR‐TKIs in EGFR‐mutant NSCLC brain metastases models, and early evidence of clinical brain metastases activity. Clin Cancer Res 22, 5130–5140. 27435396 10.1158/1078-0432.CCR-16-0399

[mol212394-bib-0002] Ceresoli GL , Reni M , Chiesa G , Carretta A , Schipani S , Passoni P , Bolognesi A , Zannini P and Villa E (2002) Brain metastases in locally advanced nonsmall cell lung carcinoma after multimodality treatment: risk factors analysis. Cancer 95, 605–612. 12209754 10.1002/cncr.10687

[mol212394-bib-0003] Choi J , Ko E , Chung HK , Lee JH , Ju EJ , Lim HK , Park I , Kim KS , Lee JH , Son WC *et al* (2015) Nanoparticulated docetaxel exerts enhanced anticancer efficacy and overcomes existing limitations of traditional drugs. Int J Nanomedicine 10, 6121–6132. 26457052 10.2147/IJN.S88375 PMC4598197

[mol212394-bib-0004] Economopoulou P and Mountzios G (2016) Non‐small cell lung cancer (NSCLC) and central nervous system (CNS) metastases: role of tyrosine kinase inhibitors (TKIs) and evidence in favor or against their use with concurrent cranial radiotherapy. Transl Lung Cancer Res 5, 588–598. 28149754 10.21037/tlcr.2016.12.06 PMC5233883

[mol212394-bib-0005] Jackman DM , Holmes AJ , Lindeman N , Wen PY , Kesari S , Borras AM , Bailey C , de Jong F , Janne PA and Johnson BE (2006) Response and resistance in a non‐small‐cell lung cancer patient with an epidermal growth factor receptor mutation and leptomeningeal metastases treated with high‐dose gefitinib. J Clin Oncol 24, 4517–4520. 16983123 10.1200/JCO.2006.06.6126

[mol212394-bib-0006] Lee YJ , Choi HJ , Kim SK , Chang J , Moon JW , Park IK , Kim JH and Cho BC (2010) Frequent central nervous system failure after clinical benefit with epidermal growth factor receptor tyrosine kinase inhibitors in Korean patients with nonsmall‐cell lung cancer. Cancer 116, 1336–1343. 20066717 10.1002/cncr.24877

[mol212394-bib-0007] Matsumoto S , Takahashi K , Iwakawa R , Matsuno Y , Nakanishi Y , Kohno T , Shimizu E and Yokota J (2006) Frequent EGFR mutations in brain metastases of lung adenocarcinoma. Int J Cancer 119, 1491–1494. 16642476 10.1002/ijc.21940

[mol212394-bib-0008] Mok TS , Wu YL , Ahn MJ , Garassino MC , Kim HR , Ramalingam SS , Shepherd FA , He Y , Akamatsu H , Theelen WS *et al* (2017) Osimertinib or platinum‐pemetrexed in EGFR T790M‐positive lung cancer. N Engl J Med 376, 629–640. 27959700 10.1056/NEJMoa1612674 PMC6762027

[mol212394-bib-0009] Omuro AM , Kris MG , Miller VA , Franceschi E , Shah N , Milton DT and Abrey LE (2005) High incidence of disease recurrence in the brain and leptomeninges in patients with nonsmall cell lung carcinoma after response to gefitinib. Cancer 103, 2344–2348. 15844174 10.1002/cncr.21033

[mol212394-bib-0010] Park SJ , Kim HT , Lee DH , Kim KP , Kim SW , Suh C and Lee JS (2012) Efficacy of epidermal growth factor receptor tyrosine kinase inhibitors for brain metastasis in non‐small cell lung cancer patients harboring either exon 19 or 21 mutation. Lung Cancer 77, 556–560. 22677429 10.1016/j.lungcan.2012.05.092

[mol212394-bib-0011] Park JW , Yun JM , Lee ES , Youn YS , Kim KS , Oh YT and Oh KT (2013) A nanosystem for water‐insoluble drugs prepared by a new technology, nanoparticulation using a solid lipid and supercritical fluid. Arch Pharm Res 36, 1369–1376. 23780798 10.1007/s12272-013-0187-2

[mol212394-bib-0012] Rho JK , Lee IY , Choi YJ , Choi CM , Hur JY , Koh JS , Lee J , Suh BC , Song HJ , Salgaonkar P *et al* (2017) Superior efficacy and selectivity of novel small‐molecule kinase inhibitors of T790M‐mutant EGFR in preclinical models of lung cancer. Cancer Res 77, 1200–1211. 28082405 10.1158/0008-5472.CAN-16-2432 PMC5334209

[mol212394-bib-0013] Togashi Y , Masago K , Masuda S , Mizuno T , Fukudo M , Ikemi Y , Sakamori Y , Nagai H , Kim YH , Katsura T *et al* (2012) Cerebrospinal fluid concentration of gefitinib and erlotinib in patients with non‐small cell lung cancer. Cancer Chemother Pharmacol 70, 399–405. 22806307 10.1007/s00280-012-1929-4

[mol212394-bib-0014] Wu YL , Zhou C , Cheng Y , Lu S , Chen GY , Huang C , Huang YS , Yan HH , Ren S , Liu Y *et al* (2013) Erlotinib as second‐line treatment in patients with advanced non‐small‐cell lung cancer and asymptomatic brain metastases: a phase II study (CTONG‐0803). Ann Oncol 24, 993–999. 23129122 10.1093/annonc/mds529

[mol212394-bib-0015] Yang KM , Shin IC , Park JW , Kim KS , Kim DK , Park K and Kim K (2017) Nanoparticulation improves bioavailability of Erlotinib. Drug Dev Ind Pharm 43, 1557–1565. 28554216 10.1080/03639045.2017.1326931

