# Supplementary figures and images for: Efficacy of nano‐particulated, water‐soluble erlotinib against intracranial metastases of EGFR‐mutant lung cancer
Source: Mol Oncol. 2018 Nov 2;12(12):2182–90. doi: 10.1002/1878-0261.12394 (PMC6275278; doi:10.1002/1878-0261.12394)

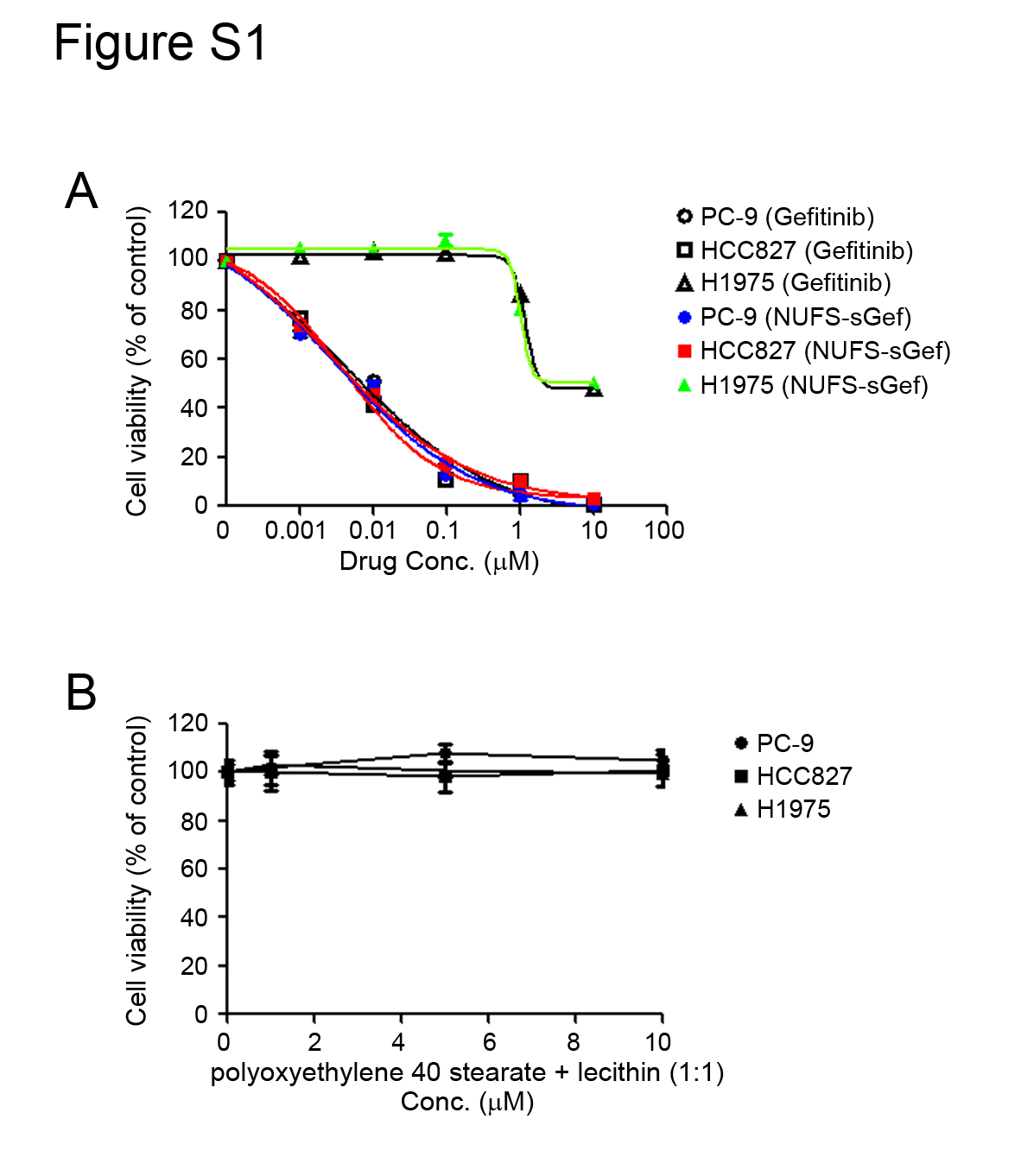

Supplement: Supplementary file 1 — Fig. S1. Effects of NUFS‐sGef in mutant‐EGFR NSCLC cells. [file MOL2-12-2182-s001.tif]
