# Supplementary material for: Efficacy of nano‐particulated, water‐soluble erlotinib against intracranial metastases of EGFR‐mutant lung cancer
Source: Mol Oncol. 2018 Nov 2;12(12):2182–90. doi: 10.1002/1878-0261.12394 (PMC6275278; doi:10.1002/1878-0261.12394)
Supplement: Supplementary file 2 [file MOL2-12-2182-s002.docx]

**Supplementary Figure legends**

**Figure S1.** Effects of NUFS-sGef in mutant-EGFR NSCLC cells. A water-soluble gefitinib (NUFS-sGef) was generated using NUFSTMtechnology. (A and B) Cells were treated with the indicated doses of NUFS-sGef, gefitinib or excipients (polyoxyethylene 40 stearate and lecithin) for72 h. Cell viability was then determined byMTT assay. Error bars are represented as mean ± SD (*n* = 3).
